# Bat origin of human coronaviruses

**DOI:** 10.1186/s12985-015-0422-1

**Published:** 2015-12-22

**Authors:** Ben Hu, Xingyi Ge, Lin-Fa Wang, Zhengli Shi

**Affiliations:** Key Laboratory of Special Pathogens and Center for Emerging Infectious Diseases, Wuhan Institute of Virology, Chinese Academy of Sciences, Wuhan, China; Program in Emerging Infectious Diseases, Duke-NUS Graduate Medical School, Singapore, 169857 Singapore

**Keywords:** Bats, SARS, MERS, Coronavirus, Emerging infectious diseases

## Abstract

Bats have been recognized as the natural reservoirs of a large variety of viruses. Special attention has been paid to bat coronaviruses as the two emerging coronaviruses which have caused unexpected human disease outbreaks in the 21st century, Severe Acute Respiratory Syndrome Coronavirus (SARS-CoV) and Middle East Respiratory Syndrome Coronavirus (MERS-CoV), are suggested to be originated from bats. Various species of horseshoe bats in China have been found to harbor genetically diverse SARS-like coronaviruses. Some strains are highly similar to SARS-CoV even in the spike protein and are able to use the same receptor as SARS-CoV for cell entry. On the other hand, diverse coronaviruses phylogenetically related to MERS-CoV have been discovered worldwide in a wide range of bat species, some of which can be classified to the same coronavirus species as MERS-CoV. Coronaviruses genetically related to human coronavirus 229E and NL63 have been detected in bats as well. Moreover, intermediate hosts are believed to play an important role in the transmission and emergence of these coronaviruses from bats to humans. Understanding the bat origin of human coronaviruses is helpful for the prediction and prevention of another pandemic emergence in the future.

## Background

Bats, with extensive geographical distribution and capability of flight, constitute the second largest group of mammalian species and have been documented as natural hosts of a large number of diverse viruses such as lyssaviruses, paramyxoviruses and filoviruses [1, 2]. In the past decade, numerous novel coronaviruses have been discovered in a wide variety of bat species throughout Asia, Europe, Africa and America [3]. Within the coronavirus genera *Alphacoronavirus* and *Betacoronavirus*, which mainly infect mammals, 7 out of the 15 currently assigned viral species have only been found in bats [4]. It is proposed that bats are major hosts for alphacoronaviruses and betacoronaviruses and play an important role as the gene source in the evolution of these two coronavirus genera [5]. Among the coronaviruses harbored by bats, some have drawn particular research interests, as they have been found to be associated with two high profile human disease outbreaks, Severe Acute Respiratory Syndrome (SARS) and Middle East Respiratory Syndrome (MERS).

In this review, we focus on the emerging coronaviruses putatively linked to a zoonotic origin from bats, represented by SARS coronavirus (SARS-CoV) and MERS coronavirus (MERS-CoV). We present an overview of current evidence for bat origin of these two viruses and also discuss how the spillover events of coronavirus from animals to humans may have happened. Considering that bats have been known to harbor more coronaviruses than any other species, it is likely that SARS-CoV and MERS-CoV won’t be the only bat coronaviruses to jump among species and cause human infections. Bat coronaviruses should be seriously regarded in light of their potential risks to public health.

### Emergence of SARS and MERS

SARS first emerged in late 2002 in Guangdong Province, southern China, as a novel clinical severe disease (termed “atypical pneumonia”) marked by fever, headache and subsequent onset of respiratory symptoms including cough, dyspnea and pneumonia. Being highly transmissible among humans, SARS rapidly spread to Hong Kong and other provinces across China and then to other 28 countries [6, 7]. By July 2003, it had caused 8096 confirmed cases of infection in 29 countries, 774 (9.6 %) of which were fatal (http://www.who.int/csr/sars/country/table2004_04_21/en/). The second outbreak in 2004 only caused 4 infections with no mortality nor further transmission [8].

The MERS epidemic emerged in the Kingdom of Saudi Arabia (KSA) since June 2012, with a similar clinical syndrome to SARS but seemingly less transmissible. In addition to respiratory illness, renal failure was identified in some severe cases [9–11]. Unlike SARS which had numerous super-spreader events, most MERS cases were independent clusters and limited to countries in the Middle East, particularly in KSA. Limited MERS cases have been reported in African and European countries and the United States of America, but exclusively in individuals travelling back from the Middle East. Some patients were reported to have a history of contact with camels while many other cases lacked this epidemiological link [9–11]. The MERS pandemic in the Republic of Korea in 2015 was caused by a single person who returned from travel in the Middle East. This made the Republic of Korea to be home to the second largest MERS epidemic with a total of 185 confirmed cases and 36 deaths [11, 12]. By 18 August 2015 a total of 1413 laboratory-confirmed cases of MERS have been reported worldwide with a median age of 50 years, including 502 related deaths. The mortality of MERS (approximately 35 %) is much higher than that of SARS (around 10 %).

### SARS-CoV and MERS-CoV represent two different species in the genus *Betacoronavirus*

#### Genomic structure and taxonomic classification

**Fig. 1:**
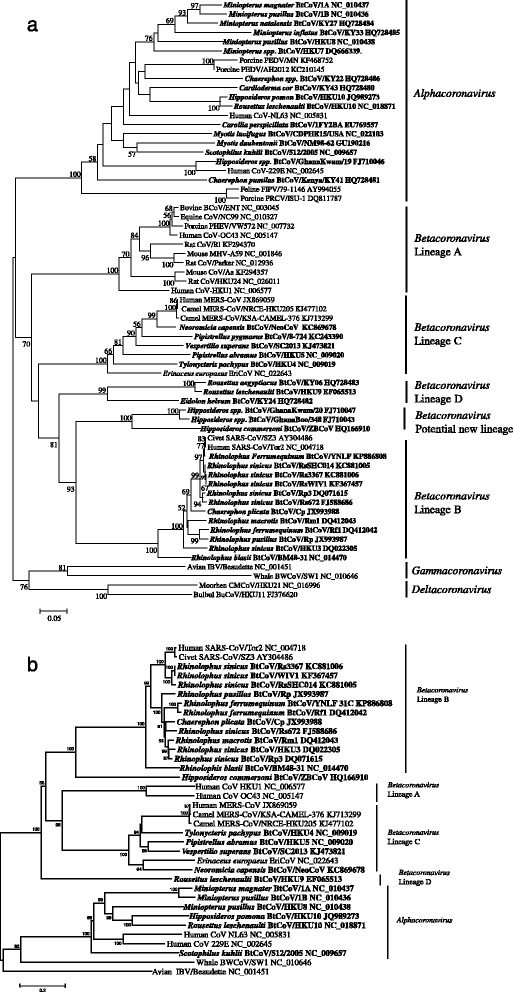
Phylogenetic analysis of bat coronaviruses with other coronaviruses. The phylogenetic tree was constructed based on 816-nt partial RdRp sequences (**a**) and full-length spike protein sequences (**b**). Available sequences were retrieved from GenBank and aligned using ClustalW. The alignment was used to construct tree by MEGA (Version 5.1) with the neighbor-joining statistical method. Bootstrap values were calculated from 1000 replicates (values ≥50 are shown). Bat coronaviruses are drawn in bold and named following bat species, plus BtCoV, strain name, and GenBank accession number

SARS-CoV and MERS-CoV share similar genome organization with other coronaviruses, but display unique genomic structures and evolutionary lineages. The coronavirus genome possesses 6-to-7 major open reading frames (ORFs) in the characteristic gene order in the 5’ to 3’ direction: ORF1a and 1b which comprise two-thirds of the genome and encode the nonstructural polyproteins, and four ORFs downstream that encode structural proteins: spike protein (S), envelope protein (E), membrane protein (M) and nucleocapsid protein (N). Some coronaviruses have a hemagglutinin-esterase (HE) gene between ORF1b and S. Besides the coronavirus-conserved genes, the SARS-CoV genome contains a number of specific accessory genes including ORF3a, 3b, ORF6, ORF7a, 7b, ORF8a, 8b and 9b [13–15]. Comparably, MERS-CoV encodes five unique accessory genes, designated ORF3, ORF4a, ORF4b, ORF5 and ORF8b. None of these genes have been shown to be related to other known coronavirus genes at the time of discovery [16, 17]. MERS-CoV was found to have 75 and 77 % amino acid (aa) sequence identity in 7 conserved replicase genes with two previously identified bat coronaviruses: BtCoV-HKU4 and BtCoV-HKU5. Based on the classification criteria of the the International Committee on Taxonomy of Viruses (ICTV), SARS-CoV and MERS-CoV represent two novel distinct coronavirus species in the genus *Betacoronavirus* (Fig. 1a and Table 1) [10, 18, 19]. Members of betacoronaviruses are separated into four lineages, A, B, C and D. SARS-CoV and MERS-CoV are clustered in lineage B and C, respectively [18].

**Table 1:** Comparison of bat coronaviruses with SARS-CoV or MERS-CoV in conserved replicase domains and structural proteins

CoV strain	Bat species	Country	% amino acid sequence identity^a^ with SARS-CoV or MERS-CoV											
			ADRP	3CLpro	RdRp	Hel	ExoN	NendoU	OMT	Concatenated domains^@^	S	E	M	N
HKU3	*Rhinolophus sinicus*	China	92.0	99.3	98.6	99.2	98.1	98.0	98.3	96.0	79.7	100	98.6	96.7
Rp3	*Rhinolophus sinicus*	China	95.4	99.7	99.5	99.7	99.2	97.4	98.3	97.7	80.3	100	97.3	98.1
Rm1	*Rhinolophus macrotis*	China	91.0	99.3	99.3	99.3	97.9	97.1	98.0	95.6	80.6	98.7	97.3	97.6
Rf1	*Rhinolophus ferrumequinum*	China	92.3	99.7	98.6	99.5	97.9	97.7	96.3	96.0	78.4	96.1	97.7	95.5
Rs672	*Rhinolophus sinicus*	China	97.0	99.3	99.8	99.3	99.1	98.6	99.0	98.4	80.2	100	98.6	98.6
Rs3367	*Rhinolophus sinicus*	China	97.0	100	99.6	99.8	99.2	98.3	98.0	98.4	92.3	100	98.2	100
RsSHC014	*Rhinolophus sinicus*	China	96.9	99.7	99.6	99.8	99.2	98.8	97.7	98.4	90.0	98.7	98.2	99.5
WIV1	*Rhinolophus sinicus*	China	97.0	99.7	99.5	99.8	99.2	98.8	98.0	98.4	92.2	100	98.2	99.8
Cp/Yunnan 2011	*Chaerephon plicata*	China	97.6	100	99.1	98.5	98.1	98.6	97.3	98.2	81.1	100	99.1	98.1
Rp/Shaanxi 2011	*Rhinolophus pusillus*	China	93.5	100	99.2	99.7	98.9	97.7	99.0	96.9	81.1	97.4	96.8	98.1
YNLF_31C	*Rhinolophus ferrumequinum*	China	97.2	99.7	99.6	99.7	99.4	98.3	97.7	98.4	79.2	100	98.6	98.3
BM48-31	*Rhinolophus blasii*	Bulgaria	76.8	94.4	98.0	98.1	95.6	91.9	91.6	88.3	75.9	92.1	91.4	88.5
HKU4-1	*Tylonycteris pachypus*	China	55.5	81	89.8	92.1	85.4	76	82.8	78.4	67	56.1	79	65.8
HKU5-1	*Pipistrellus abramus*	China	56.4	82.6	91.8	93.8	91.7	79.7	85.7	80.1	64	53.6	79	61.4
NeoCoV	*Neoromica capensis*	South Africa	86.7	96.7	98	98.4	98.2	94.1	96.3	95	64	87.7	94.2	91
SC2013	*Vespertilio superans*	China	53.5	79	88.5	93.4	85.6	76.6	88.1	85.7	69	84.5	84.7	74.4

^a^Calculated with MEGA5.1 using a pairwise deletion option; Bat SL-CoVs are listed in the upper part of the table while camel MERS-CoV and bat CoVs related to MERS-CoV in the lower part

@Seven domains were series connected and calculated with MEGA5.1 using a pairwise deletion option

ADRP, ADP-ribose 1-phosphatase; 3CLpro, coronavirus NSP5 protease; RdRp RNA-dependent RNA polymerase; Hel, helicase; ExoN, exoribonuclease; NendoU, endoribonuclease; OMT, 2’-O-methyltransferase

GenBank accession numbers: Tor2, NC_004718; HKU3, DQ022305; Rp3, DQ071615; Rm1, DQ412043; Rf1, DQ412042; Rs672, FJ588686; Rs3367, KC881006; RsSHC014, KC881005; WIV1, KF367457; Cp/Yunnan2011, JX993988; Rp/Shaanxi2011, JX993987; YNLF_31C, KP886808; EMC/2012, JX869059; HKU5-1, NC_009020; HKU4-1, NC_009019; BetaCoV/SC2013, KJ473821; NeoCoV, KC869678

#### Receptor usage

The S protein of coronaviruses is a surface-located trimeric glycoprotein consisting of two subunits: the N-terminal S1 subunit and the C-terminal S2 subunit. The S1 subunit specializes in recognizing and binding to the host cell receptor while the S2 region is responsible for membrane fusion. Compared with the S2, the S1 subunit shows much higher variability [20]. Owing to its function of receptor binding, the variation in S protein defines in large part the tissue tropism and host range of different coronaviruses [21].

Angiotensin-converting enzyme 2 (ACE2) was identified to be the functional receptor of SARS-CoV [22–24]. A 193 aa fragment (aa 318–510) of SARS-CoV S protein was demonstrated to bind ACE2 more efficiently than the full S1 domain and was defined as the receptor-binding domain (RBD) of SARS-CoV [25]. A loop subdomain (aa 424–494) that directly contacts with ACE2 was further identified as the receptor-binding motif (RBM) by crystal structure analysis [26]. In the RBM, several aa residues were found to be critical for receptor binding and changes in these key residues resulted in different binding efficiency among different SARS-CoV isolates [26–28].

Dipeptidyl peptidase 4 (DPP4, also known as CD26) was identified as a functional receptor for MERS-CoV [29] and it is relatively conserved among mammalian species. Published results indicated that MERS-CoV can infect and replicate in most cell lines derived from human, non-human primate, bat, swine, goat, horse, rabbit, civet, and camel, but not from mice, hamster, dog, ferret, and cat [29–36]. DPP4 from camel, goat, cow, and sheep can be also recognized by MERS-CoV and can support MERS-CoV replication [30, 35]. Resolved crystal structures demonstrate that DPP4-recognizing RBD is localized to the S1 C-terminal portion of S protein of MERS-CoV [37–39]. The RBD of MERS-CoV consists of ~240 residues, spanning aa 367–606, which fold into a structure consisting of two subdomains, the core subdomain and the external subdomain. The core subdomain of MERS-CoV RBD is structurally similar to that of the SARS-CoV RBD, but the external subdomain (also named as RBM) is different to that of the SARS-CoV [37–39].

### Bat origin of SARS-CoV

#### Civets are intermediate and trasnmission host of SARS-CoV

Epidemiological survey showed that early cases of SARS in 2002–2003 and all 4 cases in 2003–2004 had a history of animal contact through animal trade in wet markets or in restaurants where live animals were kept in Guangdong Province. Molecular detection and virus isolation studies suggested that the pandemic-causing SARS-CoV originated from traded civets in wet markets. This was indirectly confirmed by the massive culling of market civets, which was believed to play a major role in efficiently containing the SARS pandemics and no further SARS case was reported after 2004 [40–42].

However, subsequent extensive epidemiology studies did not find SARS-CoV in farmed or wild-caught civets, indicating that other animal(s) was involved in SARS-CoV transmission in the animal market or other trading activities and civets are unlikely the natural reservoir of SARS-CoV [43–45].

#### Discovery of diverse SARS-like coronaviruses in bats

Several years before the outbreak of SARS, two other zoonotic viruses, Nipah virus and Hendra virus, emerged in Asia and Australia and they were both known to be originated from bats [46, 47]. These led scientists to consider bats in the search of reservoirs of SARS-CoV. In 2005, a breakthrough was made as two independent research groups reported, almost simultaneously, the discovery of novel coronaviruses related to SARS-CoV in horseshoe bats (in the genus *Rhinolophus*) in China, which were termed SARS-like coronavirus (SL-CoV) [48, 49]. These bat SL-CoVs from both mainland China and Hong Kong manifested genome sequence identity of 88–90 % among themselves and 87–92 % identity to human or civet SARS-CoV isolates. The unique set of ORFs exclusively found in SARS-CoV was also present in bat SL-CoVs, demonstrating the close phylogenetic relationship between SARS-CoV and SL-CoV. The discovery of bat SL-CoV boosted researchers’ interest in coronavirus surveillance studies in bats. In following years, SL-CoV RNA was detected in *Rhinolophus* species of a wider geographic range in China. The provinces or regions where SL-CoV-positive bats were captured included Hong Kong, Guangxi, Hubei, Shandong, Guizhou, Shaanxi and Yunnan [50–53]. 7 conserved replicase domains in orf1ab of these SL-CoVs found in China were compared with those of SARS-CoV (Table 1). They all shared higher than 95 % aa sequence identity with SARS-CoV in the concatenated domains and therefore can be considered to belong to SARS-CoV species [54].

SL-CoVs were also discovered in rhinolophids from Slovenia, Bulgaria and Italy in Europe [55–57]. These European SL-CoVs exhibited significant genetic variation from Chinese isolates. The strain BM48-31 from *Rhinolophus blasii* in Bulgaria was highly divergent from Chinese isolates, displaying major sequence differences in several genes including ORF3b and ORF6 and lacking the coding region of ORF8 in its genome [55]. In Africa, novel betacoronaviruses related to SARS-CoV have been detected in *Hipposideros* and *Chaerophon* species from Ghana, Kenya and Nigeria. However, compared with Asian and European SL-CoVs, these viruses of non-rhinolophid origin were phylogenetically distant to SARS-CoV. The Western African isolates even formed a potential new lineage of *Betacoronavirus* in the phylogenetic tree (Fig. 1a) [58–60].

#### Most related ancestor of SARS-CoV in bats

Although the aforementioned bat SL-CoVs showed high sequence identity to SARS-CoV, two deletions were present in the RBM of their S proteins [48, 49]. The differences in RBM substantially changed the receptor usage. In a study using an HIV-based pseudovirus system and cell lines expressing human, civet, and horseshoe bat ACE2 molecules, the bat SL-CoV Rp3 S protein demonstrated its inability to use ACE2 as cell receptor [61]. However, the chimeric Rp3 S protein carrying the RBD of SARS-CoV S protein was conferred the capability of cell entry via human ACE2 [61]. These results suggested that bat SL-CoVs such as Rp3 were unlikely to cause human infection. Therefore, they may not be considered as the direct progenitor of SARS-CoV. Besides, the theory of bat origin of SARS-CoV lacked a powerful support due to the failure of direct isolation of SL-CoV from bats, despite numerous trials by our group as well as many others around the world.

During our longitudinal surveillance at a *Rhinolophus sinicus* colony in Yunnan Province over the years, a major breakthrough came in 2013 when diverse SL-CoVs were discovered in the single colony [53]. In this colony, there were at least 7 different strains related to SARS-CoV, HKU3, Rs672 or Rf1, based on analysis of the region corresponding to SARS-CoV RBD. Intriguingly, unlike all previously described SL-CoVs, two strains, designated Rs3367 and RsSHC014, did not contain the deletions in this region. Rs3367 showed a particularly high sequence identity to SARS-CoV in RBD and was identical to SARS-CoV in several key amino acid residues known to be important for receptor binding [53]. Whole genome sequencing revealed that Rs3367 and RsSHC014 shared more than 95 % genome sequence identity with human and civet SARS-CoV, which was remarkably higher than that of any other bat SL-CoV (76 to 92 %). Regarding individual genes, the amino acid sequence identity between Rs3367 or RsSHC014 and SARS-CoV was higher than 96 % in ORF1a, 1b, 3a, 3b, E, M and N genes [53]. Most importantly, a live SL-CoV was isolated for the first time from bat fecal samples [53]. This virus, termed WIV1, had almost identical sequence (99.9 %) to Rs3367 and was demonstrated to use ACE2 molecules from humans, civets and Chinese horseshoe bats for cell entry. It also displayed infectivity in cell lines from a broad range of species including human, pig, and bat. Furthermore, the close relatedness between WIV1 and SARS-CoV was confirmed by neutralization effect of convalescent SARS patient sera on WIV1 [53]. The isolation of a bat SL-CoV genetically closely resembling SARS-CoV and having a functional S protein capable of using the same ACE2 receptor as SARS-CoV provided robust and conclusive evidence for the bat origin of SARS-CoV.

#### Possible origin of SARS-CoV from recombination of different SL-CoVs

Despite the fact that Rs3367 or WIV1 is unprecedently close to SARS-CoV in terms of RBD region and genome identity, still there are gaps between them and the immediate ancestor of SARS-CoV. ORF8 is a highly variable gene and remarkable differences can be observed among SARS-CoVs and SL-CoVs of different host origins. Isolates from civets and from early phase of the 2002/2003 pandemic contained a single long ORF8, while in the human SARS-CoV isolates from the middle and late phase of the pandemic the ORF8 was disrupted into two ORFs, ORF8a and ORF8b, as a result of the acquisition of a 29-nt deletion after interspecies transmission to humans [8, 40, 62]. The SL-CoVs from *Rhinolophus sinicus*, including Rs3367, however, had a single ORF8 with only 32–33 % amino acid identities to that of civet SARS-CoV. In contrast, the ORF8 of two novel SL-CoV strains recently reported in Yunnan from another rhinolophid species, *Rhinolophus ferrumequinum*, exhibited exceptionally high (81.3 %) amino acid identity to civet SARS-CoV SZ3 [63]. This is consistent with isolate Rf1, a SL-CoV reported earlier from *R. ferrumequinum* in Hubei Province, of which the ORF8 shared 80.4 % amino acid identity to SZ3 [48]. Potential recombination sites were identified around the ORF8 region between SL-CoVs from *R.sinicus* and *R.ferrumequinum* and it has been suggested that the ancestor of civet SARS-CoV probably acquired ORF8 from *R.ferrumequinum* SL-CoVs by recombination [63].

### Animal origins of MERS-CoV

As with SARS-CoV, most early MERS cases had contact history with animals, e.g., dromedary camels [64, 65]. MERS-CoV RNA was detected in camels from Saudi Arabia, Qatar and Egypt and showed high similarities (>99 %) to human MERS-CoV in genomic sequences [66–71]. Serological evidence further confirmed a high prevalence of MERS-CoV infections in camels in the Middle East [72–77], Africa [78–80] and Europe (Spain) [73]. The neutralization antibodies in camels could be traced back to 1983 [73, 80]. These results strongly suggested that MERS-CoV infection in humans were transmitted through close contact with infected camels [66, 76, 81–83].

### Bat viruses related to MERS-CoV

Prior to the emergence of MERS-CoV, a group of bat coronaviruses had been reported including *Tylonycteris bat coronavirus HKU4* (BtCoV-HKU4) in *Tylonycteris* bats and *Pipistrellus bat coronavirus HKU5* (BtCoV-HKU5) in *Pipistrellus* bats in China [50, 84, 85], E.isa/M/Spain/2007 in *Eptesicus isabellinus* bats in Spain [86] and N.noc/VM366/2008/NLD in *Pipistrellus pipistrellus* bats in the Netherlands [87]. Based on genomic sequence analysis, these bat coronaviruses were grouped into lineage C of the genus *Betacoronavirus*. After the outbreak of MERS, MERS-CoV related coronaviruses were found in more bat species and countries [88–96]. Among these viruses, full-length or near full-length genomes of BtCoV-HKU4, BtCoV-HKU5, SC2013 and NeoCoV have been characterized. By genomic analysis of lineage C betacoronaviruses, MERS-CoV derived from camels show high similarities to human MERS-CoV with >99.5 % nt identities, confirming that the human and camel isolates belong to the same coronavirus species. Bat HKU4, HKU5, NeoCoV and SC2013, shared 69.8, 70, 85.6 and 75.6 % nt identities with MERS-CoV at genomic level, respectively. Seven conserved replicase domains in orf1ab of MERS-CoV related viruses were compared with MERS-CoV (Table 1). The concatenated translated domains of NeoCoV shared 95 % aa sequence identity with MERS-CoV and it could be classified as the same MERS-CoV species [54]. Other bat coronaviruses, HKU4, HKU5 and SC2013, could be considered as different coronavirus species. The most recent ancestor analysis speculated that MERS-CoV may have jumped from bats to camels approximately 20 years ago in Africa, with camels then being imported into the Arabian Peninsula [92], while HKU5 and MERS-CoV may have diverged from their common ancestor about 400 to 500 years ago [85].

Although NeoCoV is closer to MERS-CoV than other bat coronaviruses at genomic level, the phylogenetic analysis of the spike protein showed that HKU4 is the most closely related to MERS-CoV among all currently known bat coronaviruses, sharing 67 % sequence identity (Fig. 1b). This is correlated with the capability of HKU4 of using DPP4 as its functional receptor. However, HKU4 preferred bat DPP4 over human DPP4, whereas MERS-CoV showed the opposite trend [97]. It was suggested that MERS-CoV ancestors had been circulating in bats for very long time. MERS-CoV has evolved to adapt to use human receptor and the DPP4-recognizing bat coronaviruses like HKU4 may follow up, thereby posing a serious risk to human health [97, 98].

### Comparison of transmission of MERS-CoV and SARS-CoV

Both SARS-CoV and MERS-CoV are emerging zoonotic pathogens that crossed the species barriers to infect humans [10, 53, 99]. Evidence showed that SARS-CoV and MERS-CoV originated from bats, the nature reservoirs, then transmitted to human via intermediate hosts civets and camels, respectively [10, 40, 53, 81, 100]. Human SARS-CoV infection originated from the direct contact between humans and civets in markets or restaurants. Closing wet markets and cleaning civet cut off the spread chain of SARS-CoV and effectively ended the SARS epidemic [40, 42, 101]. In contrast, MERS-CoV is believed to have existed in camels for a very long time and camels are widely distributed in Middle East and African countries, serving as important transport vectors and sources of meat and milk for the local population. Therefore, it is difficult to adopt the same strategy of SARS-CoV control in the prevention of future MERS-CoV outbreaks. Until a comprehensive approach is found, which most likely will involve the effective vaccination of camels against MERS-CoV among other measures, it is envisaged that sporadic human infection will persist for some time in the future [11, 70].

### Bat coronaviruses and human coronavirus 229E (HCoV-229E) and NL63 (HCoV-NL63)

HCoV-229E was found in the 1960s and causes comparatively mild common colds worldwide [102]. A bat coronavirus detected in *Hipposideros caffer ruber* in Ghana termed Hipposideros/GhanaKwam/19/2008 was genetically related to HCoV-229E. Its RdRp fragment shared 92 % nucleotide sequence identity with HCoV-229E and they were predicted to share a most recent common ancestor (MRCA) only 200 years ago [58]. A recent study characterized more 229E-related coronaviruses discovered in hipposiderid bats from Ghana on full genome level. These bat coronaviruses were more diversified and formed a single viral species with HCoV-229E. Interestingly, phylogenetic analysis revealed the intermediate position of a 229E-related alpaca virus between bat and human viruses. These findings suggested the ancestral origin of HCoV-229E in hipposiderid bats and the role of camelids as potential intermediate hosts was hypothesized [103].

HCoV-NL63 was first isolated from babies suffering of pneumonia and bronchiolitis in 2004 [104]. To date, HCoV-NL63 has been found worldwide with up to 9.3 % detection rate in hospitalized respiratory tract samples [105]. In 2010, a bat coronavirus termed ARCoV.2 (Appalachian Ridge CoV) detected in North American tricolored bat (*Perimyotis subflavus*) in the US showed close relationship with HCoV-NL63. The MRCA for HCoV-NL63 and ARCoV.2 was predicted to have existed 563 to 822 years ago [106, 107]. Further analysis indicated that HCoV-NL63 can replicate in cell lines derived from the lungs of tricolored bats [107]. These results suggest that prototypes of HCoV-NL63 may also exist in bats and there may also be a bat origin of this human coronavirus.

## Conclusions

Although the study of bat-borne coronaviruses has only started just about 10 years ago, the scientific community has already learnt a great deal of useful lessons which will be instrumental in mitigating, predicting, and preventing future zoonotic coronavirus outbreaks. Some of these lessons are summarized below.

Bats harbor coronaviruses with great genetic diversity. It is believed that most, if not all, currently circulating alphacoronaviruses and betacoronaviruses in different mammals are evolutionally linked to ancestral coronaviruses originated from bats. Different species of rhinolophid bats in China carry genetically diverse SARS-like coronaviruses, some of which are direct ancestors of SARS-CoV and hence have the potential to cause direct interspecies transmission to humans. Meanwhile, different coronavirus species closely related to MERS-CoV are circulating in bats. Bats are likely natural reservoirs of MERS-CoV or an ancestral MERS-like CoV. It is hypothesized that bat MERS-like CoV jumped to camels or some other as yet unidentified animal several decades ago. The virus evolved and adapted with accumulating mutations in camels and then was transmitted to humans very recently. It took almost a decade from the first discovery of SL-CoV in bats to the final isolation of the SARS-CoV ancestral virus from bats, so continuing surveillance is vital to uncover the origin of MERS-CoV and bats should certainly be a priority of research. Besides, as the spike protein and host receptor are key factors of cross-species transmission of coronaviruses, characterization of the receptor and key binding sites of the spike protein will be important in estimating host tropism of bat coronaviruses and predicting spillover risk.

With human activity increasingly overlapping the habitats of bats, diseases outbreaks resulted from spillover of bat coronaviruses will continue to occur in the future despite the fact that direct transmission of bat coronaviruses to humans appears to be rare. To better prepare ourselves in predicting and preventing the next emergence of a coronavirus disease, it is necessary to maintain our vigilance in long-term coronavirus surveillance studies in bats as well as in other wildlife and livestock. Combined with other laboratory-based studies such as receptor specificity, pathogenesis and animal infection, a focus on continued surveillance will help us to improve risk assessment as well as to reveal the potential intermediate hosts that may play an important role in the interspecies transmission of various known and as yet unknown bat coronaviruses.

## Abbreviations

SARS: Severe acute respiratory syndrome
MERS: Middle East respiratory syndrome
SARS-CoV: Severe acute respiratory syndrome coronavirus
MERS-CoV: Middle East respiratory syndrome coronavirus
ORF: Open reading frame
ACE2: Angiotensin-converting enzyme 2
RBD: Receptor-binding domain
RBM: Receptor-binding motif
DPP4: Dipeptidyl peptidase 4
SL-CoV: SARS-like coronavirus
MRCA: Most recent common ancestor
